# Metal piracy by *Neisseria gonorrhoeae* to overcome human nutritional immunity

**DOI:** 10.1371/journal.ppat.1011091

**Published:** 2023-02-02

**Authors:** Ian K. Liyayi, Amy L. Forehand, Jocelyn C. Ray, Alison K. Criss

**Affiliations:** Department of Microbiology, Immunology, and Cancer Biology, University of Virginia, Charlottesville, Virginia, United States of America; Duke University School of Medicine, UNITED STATES

The bacterium *Neisseria gonorrhoeae* causes the sexually transmitted disease gonorrhea. *N*. *gonorrhoeae* is an obligate human pathogen that colonizes mucosal surfaces of the urogenital tract, pharynx, rectum, and conjunctiva, where it stimulates robust neutrophil recruitment. Successful infection requires *N*. *gonorrhoeae* to overcome nutritional immunity, the process by which hosts starve microbes of essential metals such as iron and zinc. *N*. *gonorrhoeae* has unique ways to subvert nutritional immunity, particularly by producing transporters that bind and extract metals from human metal-sequestering proteins. Because of the importance of metal acquisition to *N*. *gonorrhoeae* colonization and infection, its metal acquisition systems are potential targets for vaccines and therapeutics to combat the rise in antibiotic-resistant gonorrhea [1]. Here, we review the nutritional immunity challenges faced by *N*. *gonorrhoeae* with a particular focus on iron and zinc, how *N*. *gonorrhoeae* overcomes nutritional immunity for successful infection, and open questions for future investigation. Due to space constraints, we are not including work from other microbial systems in which nutritional immunity has been investigated; we direct the reader to recent reviews on this topic, as well as a more comprehensive review on metal homeostasis in pathogenic *Neisseria* [2–4].

## 1. What are the nutritional immunity challenges that *N*. *gonorrhoeae* faces in its obligate human host?

Free metals are scarce in humans and other animals. Instead, metals are bound by soluble proteins and compounds whose abundance varies at different locations in the host. Of relevance to *N*. *gonorrhoeae*, cervical secretions contain calprotectin (S100A8/A9) and psoriasin (S100A7) [5]. These proteins bind free zinc, limiting its availability to *N*. *gonorrhoeae*; calprotectin also binds manganese, copper, nickel, and iron [6]. Neutrophils contain abundant calprotectin [7] and the iron-binding protein lactoferrin [8]. Neutrophils also make lipocalin-2, which binds to siderophores, small iron-binding molecules released by some non-*Neisserial* bacteria [9]. Neutrophil metal-sequestering proteins are released into phagosomes and extracellularly, including in neutrophil extracellular traps [10]. *N*. *gonorrhoeae* that is exposed to serum/blood, whether in inflammatory transudate or menstrual fluid, or during disseminated infection, faces high concentrations of the iron-binding proteins transferrin and hemoglobin [11]. In contrast, seminal fluid is high in zinc [12]. Altogether, *N*. *gonorrhoeae* experiences varied metal availability when infecting humans.

## 2. How does *N*. *gonorrhoeae* sense and respond to metal limitation?

**Fig 1:**
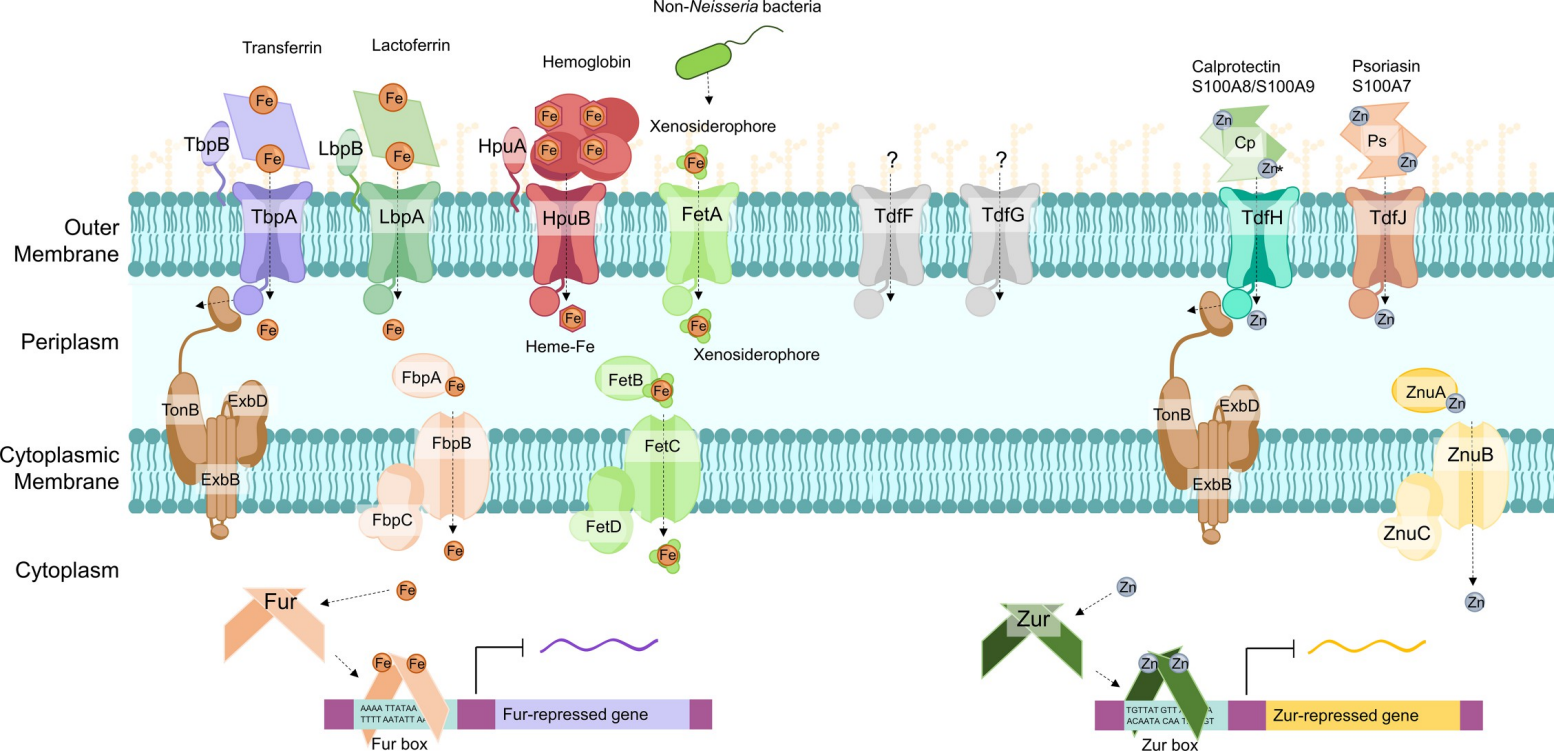
Metal acquisition systems expressed by *N*. *gonorrhoeae* in metal-limiting conditions. Outer membrane Tdfs produced by *N*. *gonorrhoeae* and their respective ligands, to date, are represented above in matching colors. Transporter–ligand interactions are described in the text. Metal ions are represented as spheres and labeled as Fe for iron and Zn for zinc. Zn* represents Zn and other divalent metals that bind at calprotectin’s His_6_ site. The TonB system energizes Tdfs to internalize metal ions by moving the occluding plug domain (periplasmic circle attached to each transporter). ABC metal transport systems associated with Tdfs are found in the periplasm and the cytosolic membrane. Cytoplasmic metalloregulators Fur and Zur bind iron and zinc, respectively, and alter expression of metal acquisition genes.

Pathogenic *Neisseria* encode the transcriptional regulators Fur and Zur, which mediate changes in expression of metal homeostasis genes in response to intracellular levels of iron [13] and zinc [14], respectively **(Fig 1)**. When intracellular iron concentration is high, Fur binds iron and dimerizes, increasing its affinity for palindromic A/T rich sequences found in the promoters of iron-responsive genes to repress their transcription. When intracellular iron concentrations decline, iron dissociates from Fur, Fur affinity for the Fur binding sites decreases, and repression of Fur-responsive genes is relieved. Similarly, Zur binds zinc and recognizes a different consensus DNA sequence in Zur-regulated genes. Genes in the *fur* and *zur* regulons encode proteins involved in metal acquisition, metal transport, and metabolism, and as-yet uncharacterized proteins [13,14]. Fur can also enhance gene expression, potentially by opening DNA for binding of RNA polymerase or other regulators [15].

Some genes in the *fur* and *zur* regulons encode ABC family metal importers, powered by ATP hydrolysis, that localize to the cytoplasmic membrane and partner with high-affinity periplasmic metal-binding proteins **(Fig 1)**. Ferric iron is bound by FbpA in the periplasm and is imported into the cytosol by the FbpB permease, energized by the FbpC ATPase [16]. Similarly, zinc is bound by the periplasmic ZnuA and imported into the cytosol via the ZnuB permease and ZnuC ATPase [17]. ZnuABC is also known as MntABC and is reported to enable manganese import in *N*. *gonorrhoeae*, to confer resistance to reactive oxygen species [18].

## 3. How does *N*. *gonorrhoeae* use outer membrane receptors for human metal-sequestering proteins to overcome nutritional immunity?

*N*. *gonorrhoeae* has the remarkable ability to produce outer membrane transporters that serve as receptors for the human metal-sequestering proteins described above, from which the transporters extract and import the cognate metals **(Fig 1)**. The transporters show specificity for the human versions of these proteins, reflecting the exquisite adaptation of *N*. *gonorrhoeae* for the human host. Importantly, the transporters can also import unbound metals, although there may not be free bioavailable metal in the host.

The prototype system in *N*. *gonorrhoeae* is the transferrin receptor, comprised of the TbpA transporter and the TbpB lipoprotein that increases the affinity of TbpA for transferrin. Structural analysis of TbpA co-crystalized with human transferrin reveals the helix finger of TbpA directly extends within a cleft on the C-lobe of human transferrin to extract iron [19,20]. The outer membrane receptors are energized by the proton motive force harnessed by TonB, ExbB, and ExbD, which pull on the “plug domain” to physically open each receptor to enable transport; hence, they are collectively known as “Tdfs,” for TonB-dependent family members. Similarly, *N*. *gonorrhoeae* expresses LbpAB for binding and extracting iron from lactoferrin, and HpuAB for hemoglobin utilization [21,22]. For zinc acquisition, *N*. *gonorrhoeae* extracts zinc from human calprotectin and psoriasin, using TdfH and TdfJ, respectively [10,23–25]. Zinc-loaded calprotectin supports zinc-dependent growth of *N*. *gonorrhoeae* in metal-stripped chemically defined medium; conversely, calprotectin sequesters zinc in metal-replete tissue culture medium such that *N*. *gonorrhoeae* growth is TdfH-dependent [10,26]. While *N*. *gonorrhoeae* does not produce siderophores, FetA enables the uptake of siderophores made by other microbes, and FetBCD drive import into the cytosol [27]. Other transporters such as TdfF and TdfG are expressed by *N*. *gonorrhoeae* in iron-limiting conditions, but their ligands are not defined [28].

## 4. How do *N*. *gonorrhoeae* responses to metal limitation enable infection?

Genes encoding metal acquisition proteins are derepressed in vivo, indicating that *N*. *gonorrhoeae* experiences metal limitation during infection [29]. *N*. *gonorrhoeae* survives in metal-limiting microenvironments in vivo by exploiting human nutritional immunity proteins as metal sources. For instance, *N*. *gonorrhoeae* survives in calprotectin-rich human neutrophil extracellular traps in a TdfH-dependent manner [10], and TbpAB is necessary for initiating urethral infection in human male volunteers [30]. The iron-repressed TdfF transporter is unique to pathogenic *Neisseriae* and contributes to intracellular survival of *N*. *gonorrhoeae*, dependent on host iron availability [28]. Additional human nutritional immunity proteins may contribute to *N*. *gonorrhoeae* infection, such as calgranulin C (S100A12), from which *N*. *gonorrhoeae* acquires zinc in a TonB-independent manner; how this is achieved is currently unknown [23].

While TdfH and TdfJ are required for planktonic growth of *N*. *gonorrhoeae* in medium where zinc is sequestered by calprotectin and psoriasin, they are surprisingly dispensable for bacteria that are adherent to cells or abiotic surfaces [26]. *N*. *gonorrhoeae* exhibits distinct transcriptional profiles between adherent and planktonic states, and in zinc-replete versus deplete conditions: Genes that are significantly up-regulated in zinc-limited, adherent *N*. *gonorrhoeae* encode the periplasmic metal-binding proteins ZnuA and FbpA, as well as metabolic proteins not previously associated with metal homeostasis [26]. Thus, *N*. *gonorrhoeae* has evolved specific adaptations to respond to and resist host-mediated metal starvation.

## 5. What are the open questions to be addressed in *N*. *gonorrhoeae* nutritional immunity studies?

Areas of ongoing and future research in *N*. *gonorrhoeae* nutritional immunity include the following:

1. What are the ligands for TdfF and TdfG? Conversely, are there bacterial receptors for other S100 proteins like S100A12/calgranulin? Which transporters and metal-sequestering proteins are used by *N*. *gonorrhoeae* at the different locations it infects in humans, whether local or disseminated disease?
2. Bioavailability of nutrient metals varies in the human host (see [4]). How do other metal-responsive genes contribute to survival of *N*. *gonorrhoeae* in metal-limited conditions? Some could serve as accessory metal acquisition proteins that remain to be characterized. Others may alter *N*. *gonorrhoeae* metabolism more broadly, an area for future study.
3. *N*. *gonorrhoeae* also requires metals such as manganese, copper, and cobalt; the Zur repressor (called PerR in that report) has been reported as responsive to manganese [31]. What gene products contribute to homeostasis of those metals, and how are the genes regulated? What is the interplay between the systems that respond to different metals?
4. *N*. *gonorrhoeae* must also contend with metal intoxication, which is used as a host defense strategy. What is the *N*. *gonorrhoeae* response to metal overload, how does it differ depending on the metal, and how does it overlap with the response to other stressors, like reactive oxygen species?
5. Although the genes encoding metal-responsive proteins are mostly conserved among *N*. *gonorrhoeae*, there are strain-specific differences in their expression and regulation [10,23]. What are the molecular mechanisms underlying these differences? Similarly, the related pathogen *N*. *meningitidis* shares many of these genes with *N*. *gonorrhoeae*; how do metal acquisition systems, particularly the outer membrane transporters, contribute to meningococcal colonization and invasion? Finally, are these metal acquisition strategies conserved in commensal species of *Neisseria*, and if so, how do they enable nasopharyngeal colonization without causing disease?

Answering these questions will require multiomics technologies, imaging modalities to measure metal concentrations in different cells and tissues, advances in biochemistry and structural biology, and the robust genetics available in *N*. *gonorrhoeae*. The ensuing discoveries will shed new light on this critical aspect of *N*. *gonorrhoeae* pathogenesis and point to new targets for combating this prevalent infectious disease.
